# Co-prescription of metoprolol and CYP2D6-inhibiting antidepressants before and after implementation of an optimized drug interaction database in Norway

**DOI:** 10.1007/s00228-022-03364-5

**Published:** 2022-07-25

**Authors:** Ane Gedde-Dahl, Olav Spigset, Espen Molden

**Affiliations:** 1 Faculty of Health Sciences, Oslo Metropolitan University, P.O. Box 4 St. Olavs plass, N-0130 Oslo, Norway; 2 Department of Clinical Pharmacology, St. Olav’s University Hospital, Trondheim, Norway; 3 Department of Clinical and Molecular Medicine, Norwegian University of Science and Technology, Trondheim, Norway; 4 Department of Pharmacy, University of Oslo, Oslo, Norway; 5 Center for Psychopharmacology, Diakonhjemmet Hospital, Oslo, Norway

**Keywords:** CYP2D6, Metoprolol, Antidepressants, Drug interactions, Drug interaction database

## Abstract

**Purpose:**

To compare the co-prescription of metoprolol and potent CYP2D6-inhibiting antidepressants before and during a 10-year period after implementation of an optimized drug interaction database into clinical decision support systems in Norway.

**Methods:**

The study was a retrospective, cross-sequential nationwide analysis of drug-dispensing data retrieved from the Norwegian Prescription Database over a 1-year period before (2007) and two 1-year periods after (2012 and 2017) implementation of a drug interaction database providing recommendations on non-interacting alternative medications. Primary outcome was changes in co-prescription rates of metoprolol and the potent CYP2D6-inhibiting antidepressants fluoxetine, paroxetine, or bupropion relative to alternative antidepressants with no or limited CYP2D6 inhibitory potential. To control for potential secular trend bias, a comparison group consisting of atenolol/bisoprolol users was included.

**Results:**

The co-prescription rate of metoprolol with potent CYP2D6 inhibitors declined following implementation of the optimized database, by 21% (*P* < 0.001) after 5 years and by 40% (*P* < 0.001) after 10 years. Compared with atenolol/bisoprolol users, patients treated with metoprolol had significantly reduced likelihood of being prescribed a CYP2D6-inhibiting antidepressant in the two post-implementation periods (OR 0.61 (95% CI 0.54–0.69) and OR 0.45 (95% CI 0.40–0.51), respectively, versus OR 0.84 (95% CI 0.74–0.94) prior to implementation). Small and mostly insignificant differences in average daily metoprolol dosage were found between patients treated with the various antidepressants.

**Conclusion:**

The present study suggests that implementation of a drug interaction database providing recommendations on non-interacting drug alternatives contributes to reduced co-prescribing of drug combinations associated with potentially serious adverse effects.

**Supplementary Information:**

The online version contains supplementary material available at 10.1007/s00228-022-03364-5.

## Introduction

Drug interaction databases have been increasingly integrated with electronic health record systems and pharmacy computer systems to screen for potential drug interactions with pop-up alerts during prescription and dispensing. Documentation on the effectiveness of medication-related alerts in changing prescription practice is, however, limited [1]. A few studies have reported decreased prescription of potentially serious drug interactions after implementation of drug interaction databases into electronic health record systems in outpatient settings [2–4]. However, several studies show that there is a high rate of inappropriate overrides of drug interaction alerts [5–11]. Databases providing recommendations about alternative medications are suggested to increase drug interaction alert acceptance [12–14], but little is known about the actual impact on trends in co-prescription of interacting drug combinations over time.

The first drug interaction database integrated with clinical decision support systems in Norway included approximately 1500 interacting drug pairs. The corresponding alerts included limited supportive information and no recommendations on how to handle specific drug interactions. Concern existed that this database generated an overwhelming number of alerts, including irrelevant or insignificant alerts, leading to a high override rate. In an attempt to improve alert acceptance and drug interaction management, an optimized database was developed which provided recommendations on non-interacting alternative medications.

As comorbidity of cardiovascular disease and depression is highly prevalent [15, 16], beta-blockers and antidepressants are commonly co-prescribed in clinical practice. Metoprolol, the most common beta-blocker prescribed to patients with cardiovascular disease in Norway [17], is metabolized primarily by the cytochrome P-450 (CYP) enzyme 2D6. Concomitant use of strong CYP2D6 inhibitors can cause several-fold increase in plasma concentrations and potentially lead to serious adverse effects. The potent CYP2D6 inhibitor paroxetine has been shown to increase the biologically available dose of metoprolol about fourfold to sixfold [18–21]. Two other potent CYP2D6-inhibiting antidepressants, fluoxetine and bupropion, are expected to cause increases in metoprolol plasma concentration of the same extent [22, 23]. Contradictory evidence exists in the literature regarding the potential clinical consequences associated with concurrent use of metoprolol and CYP2D6-inhibiting antidepressants. The only published case–control study on this issue found no increase in the risk of bradycardia among older patients receiving metoprolol with paroxetine/fluoxetine as compared with fluvoxamine/citalopram/venlafaxine/sertraline [24]. A retrospective cohort study later showed that potent CYP2D6-inhibiting antidepressants were associated with greater risk of precipitating serious hemodynamic events when combined with metoprolol than antidepressants with weak CYP2D6 inhibitory potential [25], and severe bradycardia and atrioventricular block have been reported in patients treated with these drug combinations [18, 26–30]. Despite documentation of adverse clinical effects, studies have shown that metoprolol is frequently co-prescribed with potent CYP2D6-inhibiting antidepressants [31–33]. In the present study, therefore, co-prescription of metoprolol with CYP2D6-inhibiting antidepressants was selected as an index drug interaction pair to address co-prescription trends of potentially harmful drug combinations.

The aim of this study was to compare the co-prescription rate of metoprolol with potent CYP2D6-inhibiting antidepressants before and during a 10-year period after implementation of an optimized drug interaction database into clinical decision support systems in Norway.

## Methods

### Development and implementation of an optimized drug interaction database

The database originally underlying the alert system for drug interaction checking in Norwegian pharmacies and medical offices included information on severity classification, drug interaction mechanism, and the clinical consequences of the interaction, but no management advice (Table 1). Design and development of a drug interaction database with an optimized alert interface was made by the authors of this article and financed by the Norwegian Pharmacy Association. In this optimized database, advice on how to handle specific drug interactions and recommendations on non-interacting alternative medications were included (Table 1). Of specific interest to the present study, the recommendations for the co-medication of metoprolol with paroxetine/fluoxetine/bupropion are that these combinations should be avoided and replacement with alternative antidepressant agents with no or limited interaction potential (sertraline, venlafaxine, mirtazapine, mianserin, reboxetine, or vortioxetine) is recommended. Alternative non-interacting beta-blockers with the same/similar therapeutic indications (atenolol or bisoprolol) are also suggested. If the co-prescription, nevertheless, cannot be avoided, lowering the metoprolol dose is advised.

**Table 1:** Alert information content of the original versus the optimized drug interaction database

	**Original database**	**Optimized database**
Severity rating (no action needed—precautions required—should be avoided)	x	x
Drug interaction mechanism (extent of change in drug level if known)	x	x
Potential clinical consequences	x	x
Contextual information/modifying factors (e.g., dose)		x
Management recommendations (any of the following, if applicable):		x
- Alternative non-interacting drugs or drugs with limited interaction potential - Recommendations for adjustment of dose or dosing time - Recommended monitoring - Information about contraindicated combinations and combinations that should be avoided if possible		
Documented evidence (e.g., clinical trial, case report) (if applicable)		x
Supporting references (with hyperlinks to PubMed)		x

During 2008/2009, the database was integrated with the medication dispensing systems in all Norwegian pharmacies as a tool for detecting and managing drug interactions. The responsibility for maintenance and updating of the database was later transferred to the Norwegian Medicines Agency, and from 2015, the database was also integrated with the physicians’ prescription software. Except from some hospitals and nursing homes, the database is now electronically implemented at all health care levels in Norway. The database is also freely available for drug interaction checking from the Norwegian Medicines Agency website at www.legemiddelinteraksjoner.no.

### Study design and data collection

The study was a cross-sequential nationwide analysis of drug-dispensing data retrieved from the Norwegian Prescription Database (NorPD). NorPD contains information on all prescription drugs dispensed from pharmacies, but does not include drugs administered in hospitals, nursing homes, or outpatient clinics [34]. Data from all individuals being dispensed beta-blockers and antidepressants were retrospectively analyzed over a 1-year period before and two 1-year periods after implementation of the optimized drug interaction alert system: 2007 (pre-implementation period), 2012 (post-implementation period I; database implemented in the pharmacy computer system), and 2017 (post-implementation period II; database implemented in pharmacies and physicians’ electronic health record systems). The prescription data used for the study included the following information: patient unique identifier (encrypted), drug name, dispensed volume (tablet strength and package size), dispensing date, Anatomical Therapeutic Chemical (ATC) code [35], and reimbursement code as well as prescriber identification code. Patient demographics (year of birth and gender) were also available.

Individuals who filled at least three prescriptions for the same beta-blocking agent (i.e., a drug with the same ATC code in group C07A) and with at least two prescriptions of the same antidepressant agent (i.e., a drug with the same ATC code in group N06A) during any of the study periods were defined as persistent users. Prescriptions reimbursed exclusively for “cardiovascular diseases” and “mental disorders” (identified by ICPC-2/ICD-10 diagnosis codes [36]), respectively, were included.

The following drug/drug groups were subjected to further analysis: metoprolol (study drug, predominately metabolized by CYP2D6); atenolol/bisoprolol (comparator drugs, non-CYP2D6 substrates); paroxetine/fluoxetine/bupropion (potent CYP2D6 inhibitors); and sertraline/venlafaxine/mirtazapine/mianserin/reboxetine/vortioxetine (alternative antidepressants with no or limited CYP2D6 interaction potential). All other beta-blocking and antidepressive agents were simply categorized as “other beta-blockers” and “other antidepressants,” respectively.

### Exclusion

Patients receiving injectable drug formulas and subjects prescribed different beta-blockers and/or different antidepressants during the same 1-year period were excluded.

### Data analysis

Study outcomes included the proportion of beta-blocker users who were co-prescribed antidepressants, demographic characteristics, and information about the beta-blocker and the antidepressant used (i.e., type and dose). The primary outcome was changes in co-prescribing rates of metoprolol and the potent CYP2D6-inhibiting antidepressants fluoxetine, paroxetine, or bupropion relative to alternative antidepressants with no or limited CYP2D6 inhibitory potential (sertraline, venlafaxine, mirtazapine, mianserin, reboxetine, or vortioxetine) between the pre-implementation and post-implementation periods. To control for potential secular trend bias, co-prescribing in patients taking metoprolol were compared with that in users of the non-CYP2D6 substrates atenolol/bisoprolol. Odds ratios (OR) were calculated by dividing the odds for metoprolol users of being co-prescribed potent CYP2D6-inhibiting antidepressants by the corresponding odds for atenolol/bisoprolol users.

By assuming administration of one metoprolol tablet per day, the tablet strength dispensed was used as a measure of the daily metoprolol dose. An average daily dose of metoprolol was then calculated. The prescribers’ personal identifiers were applied to determine to what extent metoprolol and antidepressants were co-prescribed by the same physicians.

Differences in study outcomes between the different groups were analyzed using *t* tests and chi-square tests. All statistical analyses were performed using the SPSS software (SPSS Inc., Chicago, IL), with significance assigned at the *p* < 0.05 level.

## Results

### Patient demographics

**Fig. 1:**
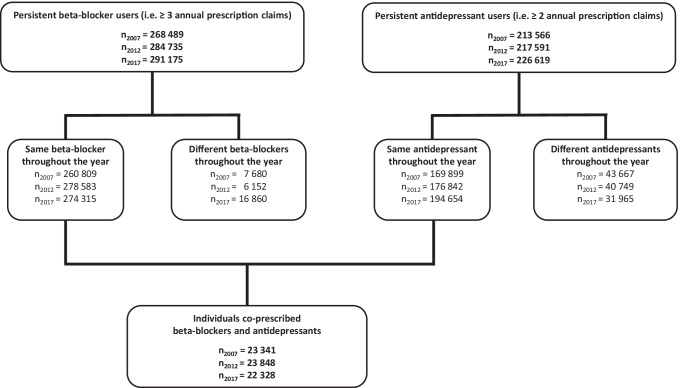
Flowchart of included patients co-prescribed beta-blockers and antidepressants in each of the three study years 2007, 2012, and 2017

A total of 23,341, 23,848, and 22,328 individuals were co-prescribed beta-blockers and antidepressants in 2007, 2012, and 2017, respectively (Fig. 1). This constituted 8–9% of all persistent beta-blocker users each year (Fig. 1). Nearly two-thirds of the study population were women, and the mean age was about 70 years (Table 2).

**Table 2:** Characteristics of beta-blocker users co-prescribed antidepressant drugs in the pre-implementation and post-implementation periods

	**Pre-implementation period (2007)** **Number (%)**	**Post-implementation period I (2012)** **Number (%)**	**Post-implementation period II (2017)** **Number (%)**
**Total**	23,341 (100)	23,848 (100)	22,328 (100)
**Mean age, years (± SD) [range]**	68.9 ± 13.3 [15–105]	70.6 ± 13.1 [18–103]	70.2 ± 13.0 [18–103]
**Gender**			
Male Female	8084 (34.6) 15,257 (65.4)	8625 (36.2) 15,223 (63.8)	8267 (37.0) 14,061 (63.0)
**Beta-blocker use**			
Metoprolol	15,658 (67.1)	17,065 (71.6)	16,421 (73.5)
*Comparator drugs*			
Atenolol Bisoprolol * Other beta-blockers*^*a*^	3636 (15.6) 760 (3.3) 3287 (14.0)	2546 (10.7) 1714 (7.2) 2523 (10.6)	1872 (8.4) 2044 (9.2) 1991 (8.9)
**Antidepressant use**			
* Potent CYP2D6 inhibitors*			
Paroxetine Fluoxetine Bupropion^*b*^	2000 (8.6) 339 (1.5) 0	1526 (6.4) 320 (1.3) 155 (0.6)	983 (4.4) 300 (1.3) 270 (1.2)
* Antidepressants with no or limited CYP2D6 inhibitory potential*			
Sertraline Mianserin Mirtazapine Venlafaxine Reboxetine Vortioxetine^*c*^ * Other antidepressants*^*d*^	2157 (9.2) 1664 (7.1) 1532 (6.6) 1447 (6.2) 15 (0.06) N/A 14,187 (60.8)	2219 (9.3) 1578 (6.6) 2332 (9.8) 1947 (8.2) 11 (0.05) N/A 13,760 (57.7)	2173 (9.7) 1424 (6.4) 3274 (14.7) 2430 (10.9) 12 (0.1) 179 (0.8) 11,283 (50.5)

*N/A* not applicable

^a^Carvedilol, labetalol, pindolol, propranolol, sotalol, timolol

^b^Bupropion was first approved for marketing in Norway in May 2007. Only 8 users qualified the inclusion criteria as persistent users of antidepressant drugs, but neither was co-prescribed beta-blockers

^c^Vortioxetine was first approved for marketing in Norway in 2013

^d^Any other antidepressant in ATC group N06A

### Co-prescription of antidepressants among beta-blocker users

Metoprolol was by far the most frequently prescribed beta-blocker, received by 67% of the study population in 2007, 72% in 2012, and 74% in 2017 (Table 2). About 18–19% of the patients were treated with either atenolol or bisoprolol (Table 2). Co-prescription of metoprolol, atenolol, and bisoprolol with different antidepressants is shown in Supplementary Table 1.

The proportion of metoprolol users prescribed CYP2D6-inhibiting antidepressants decreased gradually from 9.4% (1476/15658) in 2007 to 7.4% (1270/17065) in 2012 and 5.6% (926/16421) in 2017. Thus, the relative decline in co-prescription rate of metoprolol with potent CYP2D6 inhibitors 5 and 10 years after implementation of the optimized database was 21% (*P* < 0.001) and 40% (*P* < 0.001), respectively. The proportion of atenolol/bisoprolol users prescribed CYP2D6-inhibiting antidepressants was unchanged before and after implementation (494/4396 versus 478/4260 and 432/3916). Compared with atenolol/bisoprolol users, patients treated with metoprolol had significantly reduced likelihood of being prescribed a CYP2D6-inhibiting antidepressant, relative to alternative antidepressants, following implementation (OR 0.61 (95% CI 0.54–0.69) and 0.45 (95% CI 0.40–0.51) in the post-implementation period I and II, respectively, versus 0.84 (95% CI 0.74–0.94) in the pre-implementation period) (Table 3). Analyses of each of the CYP2D6-inhibiting antidepressant separately showed that, compared to atenolol/bisoprolol, there was a significant reduction in co-prescription of metoprolol with fluoxetine and bupropion as well as paroxetine from 2012 to 2017 (Supplementary Table 2). Both prior to and after implementation, metoprolol and the interacting drug were prescribed by the same physician in about 80% of the cases of co-prescription (data not shown). This was about the same proportion as among co-prescriptions of metoprolol with any antidepressant drug.

Table 4 shows average daily doses of metoprolol among all persistent metoprolol users with and without co-prescriptions with antidepressants. The average daily metoprolol dosage was lower in all groups of patients in 2012 compared with 2007 (*P* < 0.001). Among patients co-prescribed potent CYP2D6-inhibiting antidepressants, the average daily dose was also lower in 2017 than in 2012 (*P* < 0.01). No significant differences in average metoprolol dosage or distribution of tablet strengths between users of CYP2D6-inhibiting antidepressants and alternative antidepressants were found in 2007 or in 2012. In 2017, however, patients with potent CYP2D6-inhibiting antidepressants used a slightly lower average metoprolol dose (*P* < 0.001), and a higher percentage were prescribed a metoprolol dose of 25 mg/day (*P* < 0.001).

**Table 3:** Co-prescriptions of metoprolol and atenolol/bisoprolol with CYP2D6-inhibiting antidepressants versus antidepressants with no or limited CYP2D6 inhibitory potential before (2007) and after (2012 and 2017) implementation of an optimized drug interaction database

	**Pre-implementation period** **(2007)**			**Post-implementation period I** **(2012)**			**Post-implementation period II** **(2017)**		
	Metoprolol	Atenolol/bisoprolol	Odds ratio (95% CI)	Metoprolol	Atenolol/bisoprolol	Odds ratio (95% CI)	Metoprolol	Atenolol/bisoprolol	Odds ratio (95% CI)
Potent CYP2D6 inhibitors^a^	1476	494	0.84	1270	478	0.61	926	432	0.45
Antidepressants with no or limited CYP2D6 inhibitory potential^b^	4593	1286	(0.74–0.94) *P* = 0.0033	5886	1353	(0.54–0.69) *P* < 0.0001	7181	1519	(0.40–0.51) *P* < 0.0001

^a^Paroxetine, fluoxetine, bupropion

^b^Sertraline, mianserin, mirtazapine, venlafaxine, reboxetine, vortioxetine

**Table 4:** Daily metoprolol dosage in patients with and without co-prescribed antidepressant drugs in the pre-implementation period (2007) versus post-implementation period I (2012) and post-implementation period II (2017)

	**All metoprolol users**			**Metoprolol users co-prescribed potent CYP2D6-inhibiting antidepressants**^a^			**Metoprolol users co-prescribed alternative antidepressants**^b^			**Metoprolol users co-prescribed other antidepressants**^c^		
	**2007** (*n* = 184,134)	**2012** (*n* = 209,120)	**2017** (*n* = 212,648)	**2007** (*n* = 1476)	**2012** (*n* = 1270)	**2017** (*n* = 926)	**2007** (*n* = 4593)	**2012** (*n* = 5886)	**2017** (*n* = 7181)	**2007** (*n* = 9589)	**2012** (*n* = 9909)	**2017** (*n* = 8314)
**Daily dosage (mg) (mean ± SD)**	74.1 ± 41.8	66.1 ± 39.2*	64.1 ± 38.0*, **	72.1 ± 41.3	62.4 ± 37.6*	58.3 ± 36.1*, **, ***	71.4 ± 42.0	64.3 ± 39.1*	63.2 ± 37.6*	71.3 ± 41.3	60.9 ± 36.6*	59.6 ± 34.7*
**Distribution of metoprolol tablet strengths (%)**												
** 25 mg**	12.1%	18.6%*	19.1%*, **	12.7%	23.2%*	26.9%*, **, ***	14.5%	20.9%*	19.9%*	14.5%	23.4%*	22.1%*, **
** 50 mg**	45.5%	48.5%*	50.9%*, **	48.2%	47.2%	49.1%	47.1%	48.6%	51.2%*, **	46.6%	49.5%*	53.0%*, **
** 100 mg**	36.4%	28.6%*	26.2%*, **	33.4%	26.2%*	21.0%*, **, ***	32.5%	26.4%*	25.2%*	33.5%	23.9%*	22.3%*, **
** 200 mg**	6.0%	4.3%*	3.8%*, **	5.7%	3.4%*	3.0%*	5.9%	4.2%*	3.7%*	5.5%	3.2%*	2.7%*, **

^*^P < 0.01 vs. 2007

^**^P < 0.05 vs. 2012

^***^P < 0.01 vs. metoprolol users co-prescribed alternative antidepressants

^a^Paroxetine, fluoxetine, bupropion

^b^Sertaline, mianserin, mirtazapine, venlafaxine, reboxetine, vortioxetine

^c^Any other antidepressant in ATC group N06A

## Discussion

The present study showed that the co-prescription rate of metoprolol and potent CYP2D6-inhibiting antidepressants was significantly reduced after implementation of an optimized drug interaction database providing recommendations on alternative non-interacting drugs. Following implementation, the co-prescription rate of metoprolol and potent CYP2D6-inhibiting antidepressants decreased by 21% and 40% after 5 and 10 years, respectively.

Comparison studies on the effectiveness of drug interaction alerts in changing prescription patterns are very limited, especially in the ambulatory setting [1, 37]. A Swedish cohort study used data on dispensed prescriptions to investigate the impact of integrating the drug interaction database SFINX into the medical record system of 15 primary health care centers [4]. They found a decrease in the prescription of potentially serious drug interactions 7–10 months after implementation (RR 0.81; 95% CI 0.60–0.99), while no significant change was observed in the control group consisting of 5 other centers (RR 0.91; 95% CI 0.32–1.29).

To our knowledge, only one before-and-after study [38] has previously assessed the possible impact of implementation of a drug interaction database on co-prescription trends on a nationwide level. Our results are in accordance with that study which found that concurrent use of benzodiazepines and metabolic enzyme inhibitors decreased after implementation of a drug interaction alert system in Korea [38]. Furthermore, our results are supported by Dutch dispensing data assessing trends in metoprolol-paroxetine/fluoxetine co-prescriptions from 1999 to 2014 [32]. The trends in that study fluctuated during the observation period but decreased steadily from 2011 to 2014. Interestingly, an increase of co-prescriptions was seen the first years after one of the two drug interaction alert systems stopped signaling these interactions [32].

Compared with atenolol/bisoprolol users, patients treated with metoprolol were less likely to be prescribed a CYP2D6-inhibiting antidepressant, relative to alternative antidepressants, in all three study periods (OR 0.84; 95% CI 0.74–0.94 in 2007, OR 0.61; 95% CI 0.54–0.69 in 2012 and OR 0.45; 95% CI 0.40–0.51 in 2017). This suggests some adherence to management advice of CYP2D6-mediated drug interactions also before implementation of the optimized alert system. This view is supported by a Swedish drug register study from 2008 which showed that patients treated with paroxetine/fluoxetine were less likely to be prescribed metoprolol, relative to atenolol, compared with patients treated with citalopram/escitalopram/sertraline (OR 0.73; 95% CI 0.69–0.78) [39].

Despite the observed reduction in co-prescription rate, a relatively large number of patients were still exposed to the combinations of metoprolol and CYP2D6-inhibiting antidepressants 10 years after implementation of the drug interaction alert system. This suggests that the alerts were overridden or considered clinically irrelevant by physicians and pharmacists, consistent with the finding in a study from The Netherlands where metoprolol-paroxetine/fluoxetine combinations were still dispensed in large numbers despite drug interaction alert systems [32].

The likelihood of being co-prescribed metoprolol with a CYP2D6-inhibiting antidepressant, relative to alternative antidepressants, was significantly reduced in post-implementation period I compared with the pre-implementation period and was further reduced in post-implementation period II. In the last period, the optimized drug interaction database was integrated with the medical record system in most general practitioners’ offices as well as in pharmacies. By using data on dispensed prescriptions from a national prescription database, we do not know whether reduced dispensing of an interacting drug combination is caused by an actual decrease in co-prescribing from the physician or is a result of drug interaction management at the pharmacy. Halkin et al. reported two decades ago that dispensing of prescriptions with severe interactions was markedly reduced after implementation of a computerized drug interaction alert system in community pharmacies in Israel, while the effect on physician prescribing patterns was limited [2]. On the other hand, a Dutch study showed that non-compliance with recommendations for management of drug interactions is common among community pharmacists [40]. More recently, a study of management of metoprolol-paroxetine/fluoxetine co-prescriptions in Dutch community pharmacies revealed that less than 5% of patients prescribed these drug combinations received an alternative therapy [32].

An alternative management of the potential drug interactions between metoprolol and CYP2D6-inhibiting antidepressants is lowering the dose of metoprolol. In the present study, all patients were prescribed a mean daily metoprolol dose below 74 mg, and small and mostly insignificant differences were found between patients treated with different antidepressants (Table 4). This is in accordance with the results of Bahar et al. who found a mean daily dose of approximately 70 mg in elderly patients regardless of co-prescription with CYP2D6-inhibiting antidepressants [32]. Furthermore, the same authors found no difference in the likelihood of dose adjustment associated with concurrent use of metoprolol-paroxetine/fluoxetine as compared with metoprolol-mirtazapine or metoprolol-citalopram [41]. It is noteworthy that although the average metoprolol doses in our study were low, 25–30% of the patients treated with CYP2D6-inhibiting antidepressants received doses above 100 mg in the post-implementation periods. Metoprolol is classified as a sensitive CYP2D6 substrate and AUC values have been shown to increase fourfold to sixfold in presence of a potent CYP2D6 inhibitor [18–21].

## Strengths and limitations

The main strength of this study is that it includes the entire population of Norway, except from patients in nursing homes and hospitals, who constitute about 1% of the total. Another strength is the inclusion of relevant comparison drugs to control for potential secular trend bias.

This study also has some limitations that should be acknowledged. As only drug dispensation data are recorded in the Norwegian Prescription Database, there is no information on drug consumption. However, the study inclusion criteria of at least two to three fillings per year indicate adherence to regular treatment over time. By using the tablet strength dispensed as a measure of the prescribed daily dosage, the metoprolol doses might have been underestimated in the present study. However, other studies in Norwegian patients have reported metoprolol dosages in the same dose range. In a cohort of 18,920 patients with myocardial infarction, the maintenance metoprolol dose was 62.3 mg/day [42] and a recent prospective observational study showed a mean up-titrated dose of 68 mg/day [43].

There was no change in guidelines or treatment recommendation in Norway that could have affected the results. However, a review of the interactions between metoprolol and antidepressants with emphasis on CYP2D6 inhibition was published in the *Journal of the Norwegian Medical Association* in 2011 [22]. Here it was specified that metoprolol should not be used concomitantly with paroxetine, fluoxetine, or bupropion due to extensive interactions and the risk of serious adverse effects. This may have increased the physicians’ alertness to these interactions, and potentially have an impact on the study outcome.

## Conclusion

The present study suggests that implementation of a drug interaction database providing recommendations on non-interacting drug alternatives contributes to reduced co-prescribing of drug combinations associated with potentially serious adverse effects.

## Supplementary Information

Below is the link to the electronic supplementary material.

Supplementary file1 (DOCX 24 KB)

Supplementary file2 (DOCX 30 KB)

## Data Availability

The datasets generated and analyzed in this study are available from the Norwegian Institute of Public Health, but restrictions apply to the availability of these data, which were used under license for the current study, and so are not publicly available. Application for access to the data can be made to the Norwegian Institute of Public Health.
