# Supplementary material for: Co-prescription of metoprolol and CYP2D6-inhibiting antidepressants before and after implementation of an optimized drug interaction database in Norway
Source: Eur J Clin Pharmacol. 2022 Jul 25;78(10):1623–32. doi: 10.1007/s00228-022-03364-5 (PMC9482580; doi:10.1007/s00228-022-03364-5)
Supplement: Supplementary file 1 — Supplementary file1 (DOCX 24 KB) [file 228_2022_3364_MOESM1_ESM.docx]

**Supplementary Table 1** Co-prescription of metoprolol, atenolol, and bisoprolol with antidepressants before (2007) and after (2012 and 2017) implementation of an optimized drug interaction database

|  | **Metoprolol** | | | **Atenolol** | | | **Bisoprolol** | | | | |  |
| --- | --- | --- | --- | --- | --- | --- | --- | --- | --- | --- | --- | --- |
|  | **2007 (n)** | **2012 (n)** | **2017 (n)** | **2007 (n)** | **2012 (n)** | **2017 (n)** | **2007 (n)** | **2012 (n)** | | **2017 (n)** | | |
| ***Potent CYP2D6 inhibitors*** | | | | | | | | | | | | |
| Paroxetine | 1261 | 960 | 558 | 367 | 250 | 151 | 72 | | 135 | | 157 | |
| Fluoxetine | 215 | 209 | 181 | 48 | 39 | 40 | 7 | | 25 | | 35 | |
| Bupropion^a^ | 0 | 101 | 187 | 0 | 14 | 17 | 0 | | 15 | | 32 | |
|  | 1476 | 1270 | 926 | 415 | 303 | 208 | 79 | | 175 | | 224 | |
| ***Antidepressants with no or limited CYP2D6 inhibitory potential*** | | | | | | | | | | | | |
| Sertraline | 1427 | 1600 | 1623 | 368 | 256 | 182 | 61 | | 123 | | 177 | |
| Mianserin | 1125 | 1166 | 1092 | 253 | 148 | 95 | 56 | | 109 | | 122 | |
| Mirtazapine | 1057 | 1712 | 2483 | 213 | 219 | 221 | 61 | | 181 | | 329 | |
| Venlafaxine | 973 | 1401 | 1847 | 215 | 185 | 159 | 57 | | 128 | | 198 | |
| Reboxetine | 11 | 7 | 8 | 1 | 1 | 1 | 1 | | 3 | | 2 | |
| Vortioxetine^b^ | N/A | N/A | 128 | N/A | N/A | 16 | N/A | | N/A | | 17 | |
|  | 4593 | 5886 | 7181 | 1050 | 809 | 674 | 236 | | 544 | | 845 | |
| ***Other antidepressants*** | | | | | | | | | | | | |
| Escitalopram | 3706 | 5645 | 5231 | 691 | 714 | 575 | 192 | | 611 | | 638 | |
| Citalopram | 2653 | 2064 | 1334 | 547 | 328 | 172 | 101 | | 191 | | 171 | |
| Duloxetine | 48 | 22 | 14 | 9 | 1 | 2 | 4 | | 1 | | 1 | |
| Amitriptyline | 1590 | 872 | 686 | 418 | 139 | 89 | 78 | | 76 | | 66 | |
| Trimipramine | 694 | 602 | 463 | 203 | 96 | 57 | 31 | | 52 | | 40 | |
| Doxepin | 454 | 316 | 208 | 148 | 76 | 39 | 19 | | 26 | | 22 | |
| Clomipramine | 247 | 235 | 211 | 98 | 54 | 40 | 11 | | 23 | | 20 | |
| Nortriptyline | 85 | 71 | 89 | 20 | 11 | 9 | 5 | | 6 | | 9 | |
| Imipramine | 1 | 0 | 0 | 0 | 0 | 0 | 0 | | 0 | | 0 | |
| Moclobemide | 75 | 55 | 54 | 26 | 12 | 5 | 4 | | 5 | | 6 | |
| Fluvoxamine | 33 | 24 | 22 | 11 | 3 | 2 | 0 | | 3 | | 1 | |
| Nefazodone | 2 | 0 | 0 | 0 | 0 | 0 | 0 | | 1 | | 0 | |
| Phenelzine | 0 | 2 | 2 | 0 | 0 | 0 | 0 | | 0 | | 1 | |
| Tranylcypromine | 1 | 0 | 0 | 0 | 0 | 0 | 0 | | 0 | | 0 | |
| Agomelatine | 0 | 1 | 0 | 0 | 0 | 0 | 0 | | 0 | | 0 | |
|  | 9589 | 9909 | 8314 | 2171 | 1434 | 990 | 445 | | 995 | | 975 | |
| **Total** | **15,658** | **17,065** | **16,421** | **3636** | **2546** | **1872** | **760** | | **1714** | | **2044** | |

*N/A* not applicable

*^a^*Bupropion was first approved for marketing in Norway in May 2007. Only 8 users qualified the inclusion criteria as persistent users of antidepressant drugs, but neither was co-prescribed beta-blockers.

*^b^*Vortioxetine was first approved for marketing in Norway in 2013.
