# Supplementary material for: Co-prescription of metoprolol and CYP2D6-inhibiting antidepressants before and after implementation of an optimized drug interaction database in Norway
Source: Eur J Clin Pharmacol. 2022 Jul 25;78(10):1623–32. doi: 10.1007/s00228-022-03364-5 (PMC9482580; doi:10.1007/s00228-022-03364-5)
Supplement: Supplementary file 2 — Supplementary file2 (DOCX 30 KB) [file 228_2022_3364_MOESM2_ESM.docx]

**Supplementary Table 2** Co-prescriptions of metoprolol and atenolol/bisoprolol with paroxetine/fluoxetine/bupropion versus antidepressants with no or limited CYP2D6 inhibitory potential before (2007) and after (2012 and 2017) implementation of an optimized drug interaction database

|  |  |  | Pre-implementation period  (2007) | | |  | Post-implementation period I  (2012) | | | | | Post-implementation period II  (2017) | | | | |
| --- | --- | --- | --- | --- | --- | --- | --- | --- | --- | --- | --- | --- | --- | --- | --- | --- |
|  | | Metoprolol | | Atenolol/ bisoprolol | Odds ratio (95% CI) | | Metoprolol | | Atenolol/ bisoprolol | | Odds ratio (95% CI) | Metoprolol | | Atenolol/ bisoprolol | | Odds ratio (95% CI) |
| Paroxetine  Antidepressants with no or limited CYP2D6 inhibitory potential^a^ | | 1261  4593 | | 439  1286 | 0.80 (0.71–0.91)  *P*<0.001 | | 960  5886 | 385  1353 | | 0.57 (0.50–0.65)  *P*<0.0001 | | 558  7181 | 308  1519 | | 0.38 (0.33–0.45)  *P*<0.0001 | |
| Fluoxetine  Antidepressants with no or limited CYP2D6 inhibitory potential^a^ | | 215  4593 | | 55  1286 | 1.09 (0.81–1.41)  *P*=0.5585 | | 209  5886 | 64  1353 | | 0.75 (0.56–1.00)  *P*=0.0495 | | 181  7181 | 75  1519 | | 0.51 (0.39–0.67)  *P*<0.0001 | |
| Bupropion  Antidepressants with no or limited CYP2D6 inhibitory potential^a^ | | 0  4593 | | 0  1286 | N/A | | 101  5886 | 29  1353 | | 0.80 (0.53–1.21)  *P*=0.2960 | | 17  7181 | 34  1519 | | 0.11 (0.06–0.19)  *P*<0.0001 | |

*N/A* not applicable

^a^Sertraline, mianserin, mirtazapine, venlafaxine, reboxetine, vortioxetine

*^b^*Bupropion was first approved for marketing in Norway in May 2007. Only 8 users qualified the inclusion criteria as persistent users of antidepressant drugs, but neither was co-prescribed beta-blockers.
